# Lightfield hyperspectral imaging in neuro-oncology surgery: an IDEAL 0 and 1 study

**DOI:** 10.3389/fnins.2023.1239764

**Published:** 2023-09-18

**Authors:** Oscar MacCormac, Philip Noonan, Mirek Janatka, Conor C. Horgan, Anisha Bahl, Jianrong Qiu, Matthew Elliot, Théo Trotouin, Jaco Jacobs, Sabina Patel, Mads S. Bergholt, Keyoumars Ashkan, Sebastien Ourselin, Michael Ebner, Tom Vercauteren, Jonathan Shapey

**Affiliations:** ^1^School of Biomedical Engineering and Imaging Science, King's College London, London, United Kingdom; ^2^Department of Neurosurgery, King's College Hospital, London, United Kingdom; ^3^Hypervision Surgical Limited, London, United Kingdom; ^4^School of Craniofacial and Regenerative Biology, King's College London, London, United Kingdom

**Keywords:** hyperspectral imaging (HSI), lightfield camera, tissue differentiation, intra-operative imaging, neuro-oncology, neurosurgery

## Abstract

**Introduction:**

Hyperspectral imaging (HSI) has shown promise in the field of intra-operative imaging and tissue differentiation as it carries the capability to provide real-time information invisible to the naked eye whilst remaining label free. Previous iterations of intra-operative HSI systems have shown limitations, either due to carrying a large footprint limiting ease of use within the confines of a neurosurgical theater environment, having a slow image acquisition time, or by compromising spatial/spectral resolution in favor of improvements to the surgical workflow. Lightfield hyperspectral imaging is a novel technique that has the potential to facilitate video rate image acquisition whilst maintaining a high spectral resolution. Our pre-clinical and first-in-human studies (IDEAL 0 and 1, respectively) demonstrate the necessary steps leading to the first *in-vivo* use of a real-time lightfield hyperspectral system in neuro-oncology surgery.

**Methods:**

A lightfield hyperspectral camera (Cubert Ultris ×50) was integrated in a bespoke imaging system setup so that it could be safely adopted into the open neurosurgical workflow whilst maintaining sterility. Our system allowed the surgeon to capture *in-vivo* hyperspectral data (155 bands, 350–1,000 nm) at 1.5 Hz. Following successful implementation in a pre-clinical setup (IDEAL 0), our system was evaluated during brain tumor surgery in a single patient to remove a posterior fossa meningioma (IDEAL 1). Feedback from the theater team was analyzed and incorporated in a follow-up design aimed at implementing an IDEAL 2a study.

**Results:**

Focusing on our IDEAL 1 study results, hyperspectral information was acquired from the cerebellum and associated meningioma with minimal disruption to the neurosurgical workflow. To the best of our knowledge, this is the first demonstration of HSI acquisition with 100+ spectral bands at a frame rate over 1Hz in surgery.

**Discussion:**

This work demonstrated that a lightfield hyperspectral imaging system not only meets the design criteria and specifications outlined in an IDEAL-0 (pre-clinical) study, but also that it can translate into clinical practice as illustrated by a successful first in human study (IDEAL 1). This opens doors for further development and optimisation, given the increasing evidence that hyperspectral imaging can provide live, wide-field, and label-free intra-operative imaging and tissue differentiation.

## 1. Introduction

Approximately 4,400 people are diagnosed with a new brain tumor each year in the UK, carrying an incidence of 7 per 100,000 with malignant brain tumors accounting for 2% of all cancers in adults and accounting for 2%–3% of all cancer deaths worldwide (Robson, 2001; McKinney, 2004; Gerard et al., 2017). Despite the best advances in chemotherapy and radiotherapy, maximal safe surgical resection remains the most significant determinant of prognosis, particularly in the context of the most aggressive primary brain cancers (Sanai et al., 2011; Chaichana et al., 2013).

Many advances have been made in intra-operative tissue differentiation over the years, with adjuncts such as fluorescence-guided surgery exploiting 5-aminolevulinic acid (Stummer et al., 2006) or other dyes, and intra-operative neuro-navigation (Orringer et al., 2012) now ubiquitous within neuro-oncological surgery. Intra-operative ultrasound is also gaining increasing traction as the only widely available real-time sub-surface imaging system available in theaters (Dixon et al., 2022). Other systems, such as intra-operative MRI (iMRI) are also becoming more frequently used to guide surgeons (Rogers et al., 2021) albeit with significant cost and space implications (Dixon et al., 2022).

Despite the described advances, each of these intra-operative techniques come with their own significant limitations. Neuro-navigation accuracy is dependent on pre-operative imaging and thus once intra-operative tumor resection has commenced, accuracy can be markedly diminished (Gerard et al., 2021). The main driver of such accuracy loss is typically referred to as *brain shift* which captures the phenomenon that as mass is removed, the structure of the tissue will change and the pre-operative imaging will no longer be representative of the intra-operative situation. *Brain shift* can result in up to 1 cm inaccuracy in neuronavigation once the dura has been opened. (Henrichs and Walsh, 2014; Noh et al., 2021). Correspondingly, fluorescence-guided tumor resection cannotalways clearly differentiate tumor margins and relies upon a surgeon-led interpretation of fluorescent tissue (Schupper et al., 2021). Intra-operative ultrasound is also heavily user dependent and whilst modern systems are capable of obtaining high quality images, they require significant experience to correctly interpret, and its usability can be limited by the size of the surgical access as well as poorer image quality in older ultrasound machine models (Solheim et al., 2010; Sastry et al., 2017; Šteňo et al., 2021).

In light of the above, there is an important research emphasis on advanced intra-operative tissue differentiation techniques. This can be either point based, e.g., Raman and reflectance spectroscopy (Vaqas et al., 2018), endomicroscopy (Kakaletri et al., 2022), or wide field, e.g., multispectral (MSI) or hyperspectral (HSI) imaging (Shapey et al., 2019). HSI in particular has already been shown to be a promising tool, demonstrating the capability to facilitate real-time wide-field label-free intra-operative tissue differentiation (Fabelo et al., 2018, 2019; Shapey et al., 2019).

Hyperspectral imaging is a wide-field optical imaging technique returning spatially resolved multi-channel spectral data. Each channel corresponds to a narrow-band diffuse reflectance optical spectral measurement centered around a specific wavelength. This results in the production of a three dimensional hyperspectral image called hypercube where there are two spatial axes and a spectral axis. The diffuse reflectance spectra are determined by the optical properties of the tissues being examined, in particular their scattering and absorption properties (Jacques, 2013). There are three main approaches to the acquisition of these hypercubes: spectral scanning, spatial scanning, and snapshot imaging (Shapey et al., 2019; Clancy et al., 2020). Spectral scanning acquires all spatial pixels of a single spectral channel simultaneously, and cycles through each spectral channel in turn by utilizing spectral band-pass filters. Spatial scanning methods acquire all spectral channels simultaneously for either a single pointwise spatial location or a complete line of spatial locations, and moves through spatial locations in turn. Both spatial and spectral scanning methods measure the full hypercube, however they require long acquisition times and are prone to motion artifacts limiting their use in clinical applications. Despite evidence that these scanning approaches work in principle (Fabelo et al., 2018, 2019), intra-operative HSI systems based on them have generally not translated well to the neuro-oncology surgical workflow outside of a research capacity. This is due to limitations such as image acquisition time and the spatial footprint of the system causing significant disruption in an already time constrained and confined neurosurgical theater environment (Shapey et al., 2019; Ebner et al., 2021), albeit some advances have been made for specific use cases (e.g., in epilepsy surgery) (Pichette et al., 2016; Anichini et al., 2022). Snapshot hyperspectral imaging systems capture hypercubes in one go and can allow for real-time imaging which makes them attractive for integration in surgical practice (Shapey et al., 2019). Their implementation nonetheless leads to trade-offs in terms of spatial and spectral resolution as discussed in more as detailed further in this work.

Mosaic snapshot HSI represents the most widespread approach to snapshot hyperspectral imaging. It utilizes a sensor where each pixel has a dedicated band-pass filter, allowing collection of a single channel per spatial location in a single shot (Geelen et al., 2014). Spectral filters are typically arranged in small patches of 4 × 4 or 5 × 5 pixels repeated in a mosaic pattern across the whole imaging sensor. This configuration is equivalent to obtaining a lower spatial resolution image per spectral channel with a sub-pixel misalignment across spectral channels. The size of the patch induces the loss of spatial resolution but also dictates the number of bands that are acquired simultaneously. The raw data from snapshot mosaic HSI sensors is thus restricted in terms of both spatial and spectral resolution. Subsequent post-processing to infer the full hypercube can nonetheless mitigate the resolution losses. Spatial resolution can be improved using classical or learning-based interpolation (Li et al., 2021, 2023). Spectral correction can remove parasitic cross-talk effects due to neighboring pixels (Pichette et al., 2017). Mosaic snapshot HSI sensor combined with fast implementation of appropriate post-processing steps allows for real-time hyperspectral imaging which in turn enables non-invasive analysis of tissues to provide physiologically relevant parameters (e.g., tissue oxygenation) with the potential to provide surgical guidance (Ayala et al., 2021; Ebner et al., 2021). Building on these foundations, hyperspectral imaging systems capable of acquiring tissue spectral information whilst integrating seamlessly in to the surgical theater workflow have been demonstrated (Ebner et al., 2021; Ayala et al., 2023). However, mosaic snapshot HSI remains limited to acquiring a relatively small number of spectral bands in a relatively confined range of the optical spectrum. Being able to acquire more spectral bands in a broader range of the spectrum would provide further opportunities for HSI in surgery, enabling the extraction of imaging biomarkers currently inaccessible to mosaic snapshot HSI.

**Figure 1:**
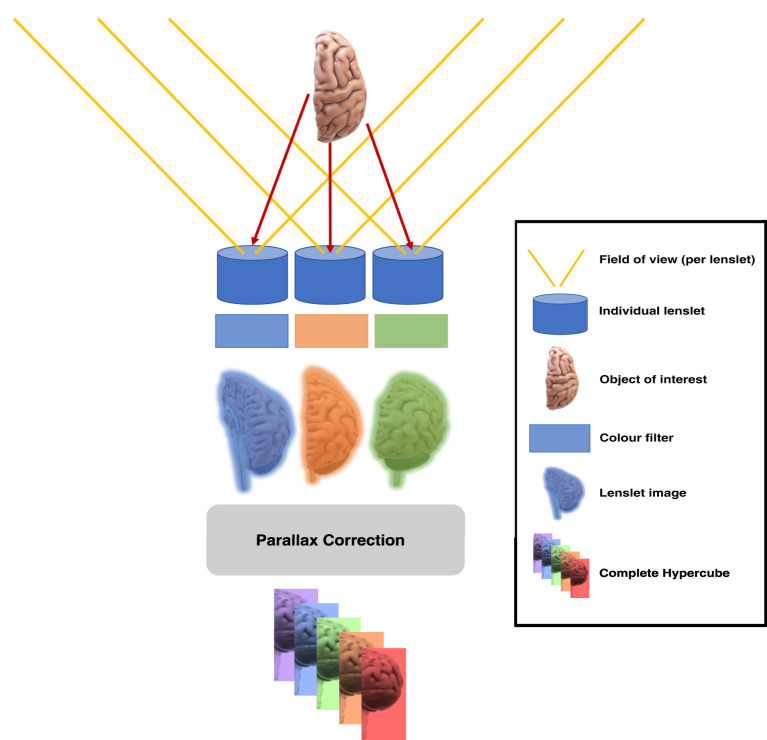
Micro-array of lenslets permits light from a single object to pass through the filters at different angles, creating different spatial and spectral perspectives of the same object.

**Figure 2:**
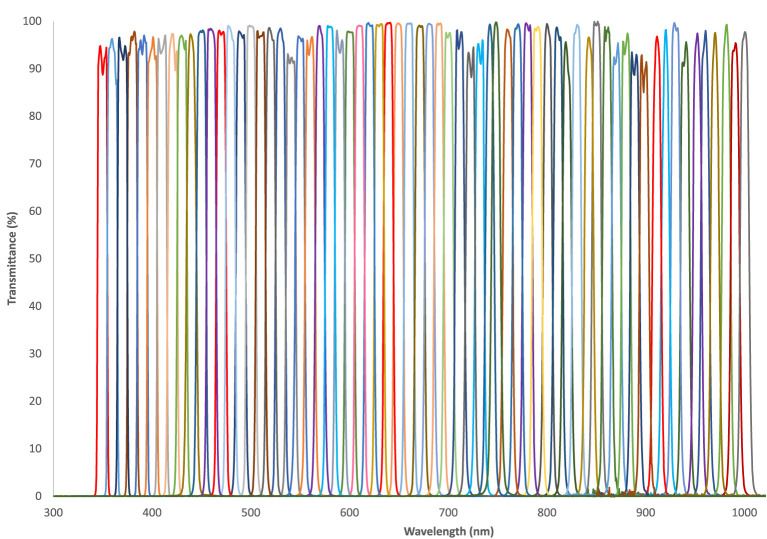
Band pass response curves for the 66 lenslet-specific filters in the Cubert Ultris ×50 Lightfield HSI camera. The central wavelength ranges from 350 to 1,000 nm, with a full width half maximum (FWHM) of 10 nm for each filter.

Lightfield hyperspectral imaging is a novel HSI method in which a large sensor is combined with a microarray of lenslets and lenslet-specific spectral filtering capabilities (Cui et al., 2020). The latter can for example be achieved with a single large continuously variable spectral filter or with an array of lenslet-specific spectral filters. With such a configuration, the sensor area covered by a single lenslet will capture data from a lenslet-specific spectral area under a lenslet-specific viewing angle as illustrated in Figure 1. Each of these lenslets views the same object from a different angles, thus facilitating the collection of radiance rays across all planes; this is the “Light Field” (Gershun, 1939; Buehler et al., 2001; Wu et al., 2017; Zhou et al., 2021). Within each lenslet image the spectral filtering properties are achieved by a different optical band pass filter for each lenslet, which will induce a subsidiary dependence of the spectral response on the pixel location in the sensor. Calibration and computational post-processing allows for the reconstruction of specific spectral bands from the collection of lenslet images. In the case of the Cubert Ultris ×50, which we use in this work, 155 spectral bands are reconstructed from the 66 lenslet images. Whilst we do not have access to the reconstruction algorithm from Cubert, the oversampling from 66 lenslet images with spatially-varying spectral characteristics to 155 fixed spectral bands provides regularly sampled spatio-spectral hypercubes with no loss of information. The band response curves for the Ultris ×50 can be seen in Figure 2.

This makes for a significant improvement in spectral resolution compared to snapshot mosaic HSI systems. Lightfield HSI provides much more refined spectral data, which may facilitate better tissue differentiation capabilities (Seidlitz et al., 2022) and can be achieved whilst maintaining the real-time imaging capabilities of snapshot mosaic HSI. Whilst the potential advantages of an intra-operative lightfield HSI system are clear, there are many challenges to developing a system that can be used in clinical practice (surgical sterility, data acquisition and validation, usability and resolution to name but a few).

In this work we have used the surgical device development framework outlined by the IDEAL (Idea, Development, Exploration, Assessment and Long-term follow-up) collaboration (McCulloch et al., 2013; Marcus et al., 2022) to describe the first clinical use of a novel lightfield hyperspectral imaging system. This system is capable of obtaining *in-vivo* spectral data from a high number of narrow spectral bands (155) across the visible and near-infrared spectral range (350–1,000 nm). Our work incorporates the design considerations set out by Ebner et al. (2021) but with the capability to acquire far greater spectral resolution, and in conjunction with those considerations set out in the IDEAL framework (McCulloch et al., 2013). To the best of our knowledge, we present the first use of a Lightfield HSI system in any clinical setting.

The remainder of this manuscript is structured as follows. Section 2 provides an overview of related work in surgical hyperspectral imaging. Sections 3 and 4 follow the structure recommended by the IDEAL collaboration (McCulloch et al., 2013; Marcus et al., 2022) with regards to the necessary steps required for both pre-clinical (IDEAL 0, Section 3) and first in human (IDEAL 1, Section 4) studies. Section 5 then builds on the findings from our IDEAL 0 and IDEAL 1 studies, providing insights into the preparation of an IDEAL 2a study with some early work being presented toward it. Finally, discussion and conclusions are provided in Sections 6 and 7.

## 2. Related works

HSI has been shown to have multiple potential applications within the medical field (Spigulis, 2017; Leon et al., 2020; Torti et al., 2020) and the surgical field (Shapey et al., 2019). Specifically within the neurosurgical literature, its reported use spans from intra-operative tissue differentiation capabilities, particularly in glioma surgery (Fabelo et al., 2018, 2019; Manni et al., 2020; Ebner et al., 2021; Anichini et al., 2022; Puustinen et al., 2023), to intra-operative visualization of cortical haemodynamic responses (Giannoni et al., 2018) including during epilepsy surgery (Pichette et al., 2016). There is also a growing body of work where HSI is used to try and quantify fluorescence (e.g., with 5-ALA) in brain tumor surgery (Valdes et al., 2019; Walke et al., 2023). This could reduce subjectivity over which regions of tissue are “fluorescing” and also detect and quantify fluorescence in low grade brain tumors (Widhalm et al., 2019; Kiesel et al., 2021; Walke et al., 2023).

Work has also been carried out to obtain reflectance information in *ex-vivo* tissue and tumor samples from brain tumor surgery with a view to creating a bank of spectra for common neurosurgical tumor types, including gliomas (Gebhart et al., 2006; Shapey et al., 2022). However, it was noted that even changes in the method of tissue preparation (fresh vs. frozen) could alter the reflectance spectra (Shapey et al., 2022) and thus the drive has been toward obtaining *in-vivo* data to further guide deep learning algorithms (Fabelo et al., 2018, 2019).

Various system designs have been considered, depending on the primary outcome of the study. For example, in a study where small changes in tissue haemodynamic response needed to be recorded, a mosaic snapshot HSI system was integrated into the surgical operating microscope, removing the issue of motion artifacts, which may otherwise obscure subtle spectral information (Pichette et al., 2016). Other systems, aiming to achieve high spatial and spectral resolution for tissue differentiation purposes, constructed standalone systems that incorporated spectral scanning HSI cameras covering a very broad spectral range (400–1,700 nm) (Fabelo et al., 2018). Whilst certainly demonstrating their capabilities within the confines of the research question asked, there were still developments to be made in terms of achieving an optimal HSI system with multi-use capabilities that integrates into the neurosurgical workflow. For example, the surgical microscope is not used in every case (e.g., convexity meningiomas) and thus an alternative, stand alone HSI system would need to be available in order to target these cases. Fabelo et al. (2018, 2019) demonstrated that their stand alone system worked very well for tissue differentiation in neuro-oncology surgery, however the significant spatial footprint of the hardware and slow image acquisition time make this a challenge to translate into regular clinical practice (Ebner et al., 2021).

These limitations led Ebner et al. (2021) to consider the design specifications for an HSI system that would be capable of meaningful data acquisition across uses, whilst translating well into the neurosurgical workflow; this ultimately led to a snapshot HSI camera coupled to a standard neurosurgical exoscope, which can be mounted with ease to the surgical operating table. This allowed for real-time data acquisition with a small spatial footprint and integrated very well into the neurosurgical workflow during their first in human study (Ebner et al., 2021).

To the best of our knowledge, there has not been any previous demonstration of a lightfield HSI system in any clinical setting.

## 3. Pre-clinical phase (IDEAL 0)

Starting from the foundations laid by Ebner et al. (2021) for the integration of hyperspectral imaging systems into the neurosurgical workflow, we used a systematic approach as per the IDEAL framework. This ensures validation and development of complex medical technologies occur simultaneously to ensure efficacy, transparency and safety (McCulloch et al., 2013; Marcus et al., 2022) in order to take our conceptualized neurosurgical lightfield hyperspectral imaging system, using the Cubert Ultris ×50 Lightfield HSI camera, to a first in-human use.

### 3.1. Device classification and risk assessment

As per the IDEAL 0 (pre-clinical) checklist, we have classified our system as a non-invasive, surgical system, which would equate to an EU regulatory class IIa or IIb system (see Appendix), https://www.medical-device-regulation.eu.

Our lightfield HSI system was then risk assessed using the Failure Models and Effects Analysis (FMEA) (Gilchrist, 1993). The results of this confirmed our system to be in the FMEA “Low Risk” category.

### 3.2. Study perspectives

The IDEAL 0 (pre-clinical) checklist then requires a classification of the study according to four perspectives: device, patient, clinician, and system. Our primary focus is on the device perspective and our pre-clinical study could therefore be classified as a device study. However, we considered all four perspectives in this work and thus report on each of these.

#### 3.2.1. Device perspective—Design

Our Lightfield HSI system consists of a number of key components, in order to acquire high quality HSI data from within the confines of the neurosurgical operating theater. These components are:

- *Lightfield HSI camera* to acquire HSI data.
- *Light guide* to deliver light from an appropriate light source to the field of view.
- *Light source* to provide optimal illumination of the field of view across a broad wavelength range.
- *Workstation and software* to process and store HSI data.
- *Display stack* to house the workstation and display HSI generated images to the surgeon.

The Cubert Ultris ×50 is a lightfield HSI camera capable of obtaining very high spectral resolution across 155 bands across the visible (VIS) and near infrared (NIR) ranges. It also has a relatively compact form factor, making it a good candidate for use in our system. The Cubert Ultris ×50 was procured as an off-the-shelf product from Cubert-GmbH.

The light guide selected was a Karl Storz 495 tip fiber optic light guide, used as standard in clinical practice for exoscopic, laparoscopic and endoscopic cases. This facilitates an even distribution and intensity of light to the region of interest.

The light source selected was the Asahi Max 350 VIS-NIR. This light source provided light with wavelengths spanning the visible and NIR spectra (350–1,000 nm), sufficient to ensure the Ultris ×50 is able to acquire broad spectral data.

A custom made graphical user interface (GUI) was created and installed onto a high-performance computer to facilitate data capture and storage. Encryption of the hard drive was used to promote cybersecurity.

A surgical grade endoscopic stack with isolation transformer power supply (Endocarts VC-480) mounted with surgical grade display monitors (Cybernet PX24) was used to house the system and display information to the surgeon.

**Figure 3:**
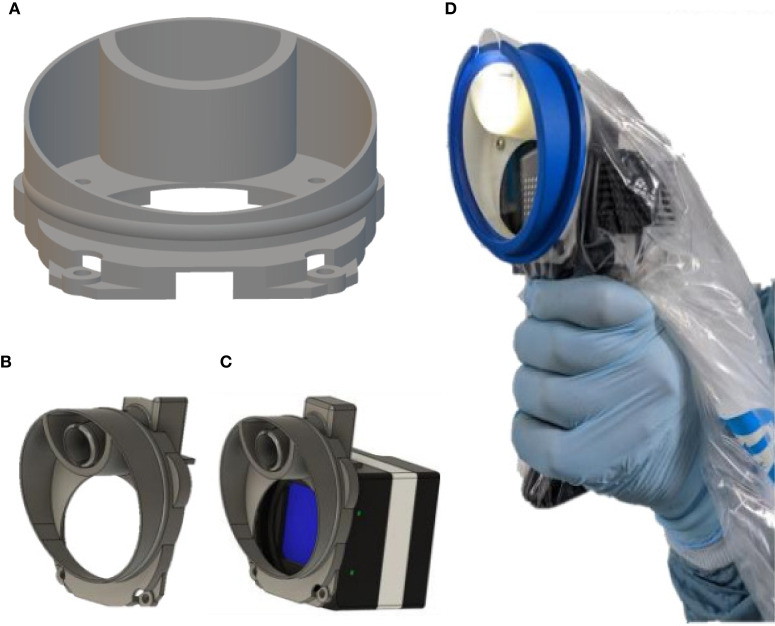
**(A)** CAD design of custom made mount for camera housing and light guide. **(B)** 3D custom printed light guide (superior aperture) and drape mount. **(C)** Graphic of mount attached to Ultris ×50 housing. **(D)** System draped for use as in neurosurgical theater.

With these core components acquired, we considered various aspects required to make the system both technically safe for use in the neurosurgical operating theater environment and capable of performing the required task. With regards to safety, there are a number of considerations to be made, the foremost of which is the certainty of surgical sterility. The Ultris ×50 is not able to be sterilized to surgical standards and thus required adaptation to facilitate draping with readily available surgical sterile drapes. In this case Leica 221-88H surgical microscope drapes were chosen. Where the possibility to have custom made drapes to suit our needs was considered, it made more practical and financial sense to adapt our system to fit with proven sterility solutions that were readily available. In order to achieve this, a computer aided design (CAD) customized 3D printed adapter and light guide mount was created and attached to the camera housing, thus allowing easy attachment of the Leica 221-88H microscope drape lens cap, without compromising sterility as shown in Figure 3. This was printed using polylactic acid (PLA) on a 3D printer with *z* resolution of 0.1 mm and *x*, *y* resolution of 0.3 mm.

In order to achieve an optimized design, several iterations of the mount were tested. Initially, the light guide was trialed parallel to the Ultris ×50 at positions 12, 3, 6, and 4 of a clock face. For simple ergonomics, the 12 O'clock position worked well, however a parallel angle did not facilitate illumination of the entire field of view and so different angles were trialed. Slight changes in these angles of the light guide and position of the optical window resulted in sub-optimal illumination of the region of interest, or internal reflections/parallax effect created by the sterile lens cover of the microscope drapes. The final design involved a central and superiorly mounted light guide with an optical window angled at 56 degrees. This provided the optimal illumination of the field with reduced parallax effect/internal reflections by creating an angle at which the light is totally transmitted through the drape lens cover and not reflected, known as Brewster's angle (Brewster, 1815; Lakhtakia, 1989). This was achieved by manually adjusting the angle until internal reflections had disappeared from the displayed images as per visual assessment.

The technical safety of each component of the system was also needed to be rigorously assessed. This was done firstly by using industry standard equipment where possible, including light source (Asahi Max 350 VIS-NIR), light guide, medical grade display monitors (Cybernet Cybermed PX-24) and imaging stack with inbuilt isolation transformer (Endocarts VC-480). All components that were not specifically approved for medical use were incorporated within the sterile surgical drapes. This led to the need to consider rigorous temperature checks in order to confirm that the components within the drapes would not exceed a temperature that could damage the surgical drapes, the user or the patient. To pre-emptively combat any issues with component temperature, our design incorporated the addition of side mounted heatsinks (RS 14 K/W foil adhesive) and a hand-held grip to facilitate use. The temperature of the system was tested whilst fully draped both with and without heatsinks and the addition of heatsinks showed a reduction of 1°C. The range was 31–48°C without heatsinks and 31–47°C with heatsinks. The maximum temperatures , measured from the camera housing, were achieved after 20 min continuous use, far longer than would be expected for a typical HSI image acquisition, which would typically be in the region of 2–3 min maximum. Although the melting point for the drapes was not specifically assessed, these temperatures were deemed to be well within the documented polyethylene film melting points of 110–150°C (Ogawa et al., 1998; Abdel-Bary, 2003). No damage to the sterile drapes or system was observed.

During mock data acquisition, we first used software designed by Cubert to run the Ultris ×50 (Cubert “Touch”), which was found to run at 1 frame per second (fps) with a latency of four seconds. This was too slow for a handheld system due to the fact that there will always be some degree of motion. Subsequently, we developed a customized graphical user interface (GUI) to allow for real-time user control of the acquisition software. The application was written in C++ using an OpenGL backend, with GLFW and ImGui for creating and controlling the display window and user interface. Due to the high bandwidth requirements of the camera, two modes of operation with low and high spatial resolution were implemented. In the first “Viewfinder” mode, 2 × 2 pixel binning of mono8 pixels is utilized to achieve a frame rate of 25 fps. This mode allows for real-time acquisition of spatially compressed data, suitable for situations demanding higher temporal resolution such as bringing the camera in focus on the region of interest. Conversely, the second “imaging” mode leverages the full sensor resolution of 7,920 × 6,008 mono12 pixels, while still achieving an interactive frame rate of 5 fps. This configuration ensures the capture of finely detailed hyperspectral images, suitable for situations that prioritize spectral fidelity over temporal resolution such as tissue characterization for decision making support. In the “imaging” mode of operation, where the full resolution of 7,920 × 6,008 mono12 pixels is utilized, each hyperspectral frame occupies 68 MB of memory. This high-resolution image data results in a data transfer rate of ~350 MB per second when capturing at an interactive frame rate of 5 fps. The substantial data throughput requires the use of a GigE Vision Ethernet connection, which efficiently handles the continuous flow of data between the hyperspectral camera and the computer. Both modes only displayed a grayscale view of the scene, rather than a color RGB reconstruction, to further reduce the computational load during intra-operative use to allow for faster and smoother operations for the user. Post-processing using the Cubert “Touch” software was then used to make full use of the captured data.

#### 3.2.2. Device perspective—Technical effectiveness

**Figure 4:**
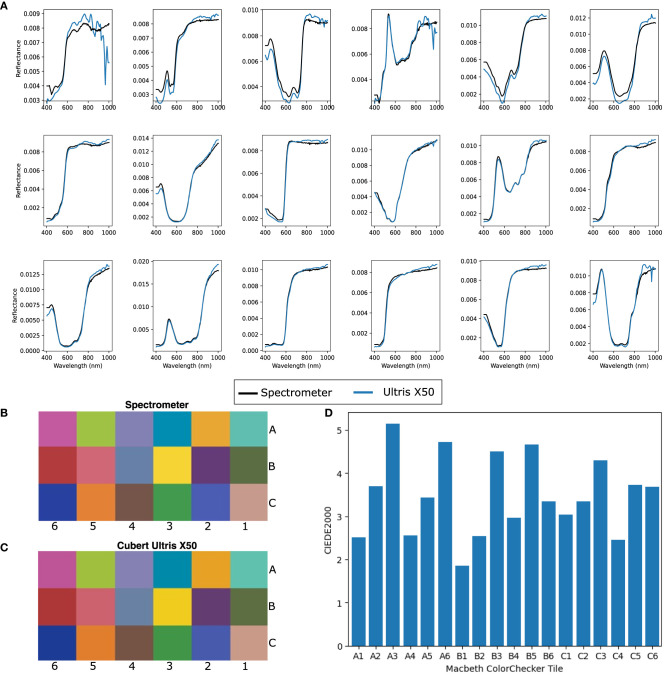
**(A)** Normalized spectra for each corresponding Macbeth ColorChecker tile. **(B)** Ground truth sRGB reconstruction of Macbeth ColorChecker tiles using specrometer measurements. **(C)** sRGB reconstruction of Macbeth ColorChecker tiles using Cubert Ultris ×50 Lightfield HSI system. **(D)** CIEDE 2000 errors per tile between spectrometer and Cubert Ultris ×50.

To assess system capability, it was first validated by using a color checkerboard (Macbeth ColorChecker) with known sRGB/CIEXYZ values and a reference spectral measurements available for each tile, which we acquired using the Ocean Optics Maya 2000 Pro high sensitivity spectrometer. HSI images of this checkerboard were acquired using the Ultris ×50 at 25 cm from the target and spectra from the central region of each colored tile were generated and plotted along with the ground truth (spectrometer) readings (Figure 4A). A standard color image was then reconstructed from the white balanced hypercube into the CIEXYZ color space, using the function outlined in Magnusson et al. (2020). CIEXYZ represents values for every perceivable color (Smith and Guild, 1931; Magnusson et al., 2020), although current display monitors are not capable of displaying color at this high fidelity. CIEXYZ values can therefore be translated into the sRGB color space by multiplying the CIEXYZ vectors by a literature 3 × 3 matrix and gamma correcting as detailed in Magnusson et al. (2020). This sRGB image can be used for direct visual comparison between the spectrometer-based color reconstructions and Ultris ×50 images. Subsequently, a square section of pixels from the central region of each color tile was selected and the spectra for each tile was averaged and converted into the sRGB color space as above. This allowed for a subjective comparison between the sRGB colors generated from the Ultris ×50 and those computed from the ground truth spectrometer measurements as seen in Figures 4B, C. In order to provide a quantitative comparison, the CIEXYZ vectors were converted to the L*a*b** color space and a Δ*E* difference measurement (CIEDE2000) was carried out as per (Sharma et al., 2005). Δ*E* values of less than two are deemed to represent color differences imperceptible to the eye, where as those under 6 are deemed acceptable in most commercial instances of color reproduction (Yilmaz et al., 2009). As seen in Figure 4A, there is clear correlation between the Ultris ×50 and the ground truth spectrometer readings, which we have represented visually in sRGB in Figures 4B, C and quantitatively with Δ*E* values in Figure 4D. The MacBeth ColorChecker provides a well characterized representative sample of colors in the real world with know spectrum. It allows for full spectral comparison and thus provides the industry standard for evaluating HSI spectral fidelity (Clancy et al., 2020). sRGB reconstructions can also provide an intuitive visual comparison of spectral quality (Wisotzky et al., 2019; Bahl et al., 2023).

**Figure 5:**
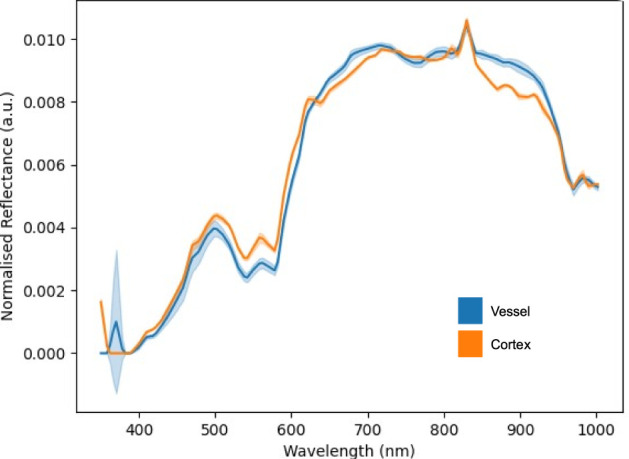
L1 normalized spectral curves of porcine vessel and cortex with standard deviations.

**Figure 6:**
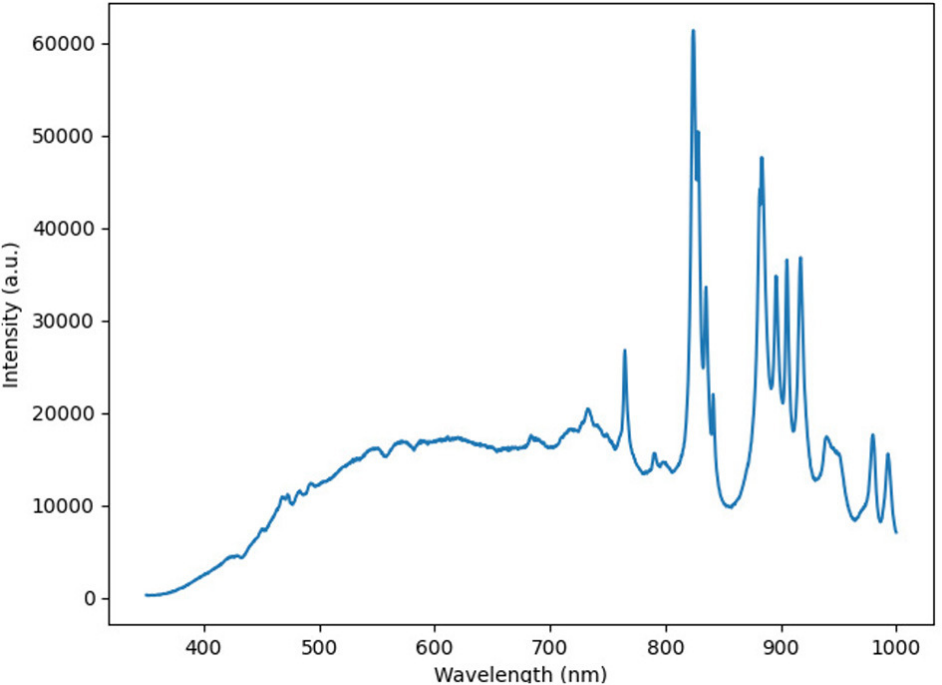
Spectra of the Asahi Max 350 VIS-NIR light source.

To evaluate our system in a more realistic scenario, a cadaveric porcine brain was imaged using our lightfield HSI system setup and spectra for cerebral cortex and vessels were acquired under simulated operating theater conditions. Our system showed clear capability in acquiring spectra for each tissue type as seen in Figure 5. This figure shows the L1 normalized spectra, i.e., each row of values is modified so that their sum is equal to 1, with standard deviations for each tissue type. As seen in Figure 5, there appear to be two abnormal spectral peaks between 800 and 850 nm wavelengths. This can be explained when considering the light source we are using (Asahi Max 350 VIS-NIR), which becomes less smooth in the higher wavelength ranges as show in Figure 6. This is an expected behavior for xenon light sources (Choudhury, 2014).

#### 3.2.3. Clinician perspective

**Figure 7:**
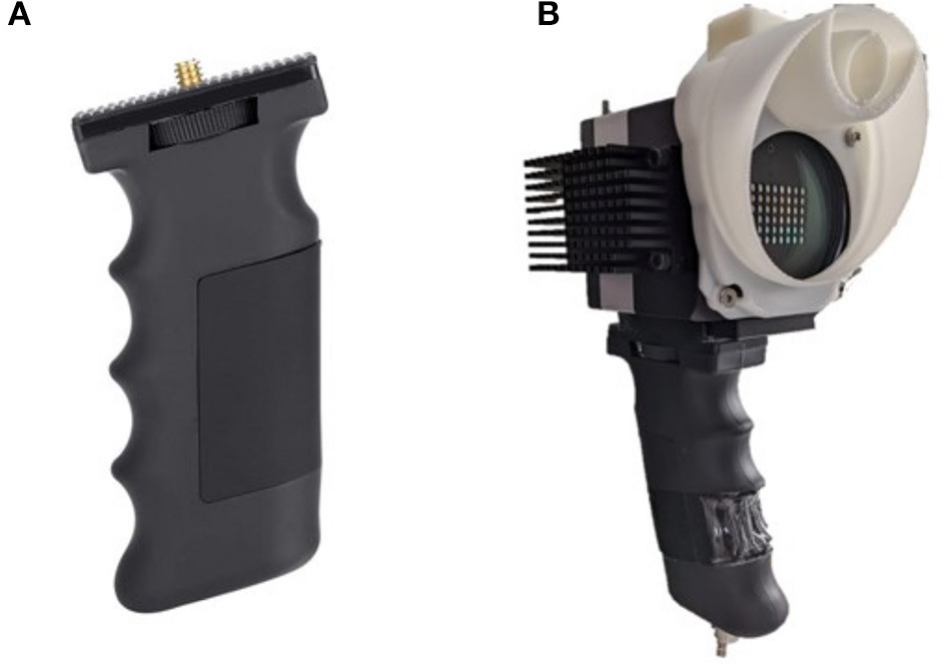
**(A)** Vbestlife camera pistol grip, portable mount. **(B)** Grip attached to Ultris ×50 along with custom printed drape and light guide mount.

To facilitate integration into the neurosurgical workflow, ergonomics and economy of movement whilst using the system, a widely available portable mount handle grip (Vbestlife camera pistol grip, Figure 7) was attached to the camera chassis via a standard 1/4 inch thread screw, leading to a handheld intra-operative system.

Two neurosurgical trainees and a consultant neurosurgeon attempted mock data acquisition using both the Cubert “Touch” software and the customized GUI. The latter was deemed subjectively much easier to use for data acquisition as there was no noticeable image lag when identifying the region of interest.

#### 3.2.4. System perspective

This work has the potential to contribute toward the increasing literature relating to the specific and individual spectral signatures of brain tumors using HSI (Fabelo et al., 2018, 2019; Anichini et al., 2022). This is crucial as brain tumor surgery still caries a 3% mortality (Williams et al., 2016) and a 3.4% average complication rate (De la Garza-Ramos et al., 2016), with an overall morbidity of up to 44.4% (Moiyadi and Shetty, 2012). Furthermore, an average of 0.5% of patients return to surgery for further tumor resection within 30 days, and up to 14% in some tumor types (Avula et al., 2013). The potential ability for HSI to clearly delineate tumor margins from healthy brain tissue (Leon et al., 2021) may significantly reduce surgical morbidity by improving the rate of maximal, safe tumor resection. (Williams et al., 2021). The methods used in this work to develop a real time, high spectral resolution surgical HSI system will impact far beyond the related research community. Importantly, this first-in-human use of a safe intra-operative lightfield HSI system to successfully characterize different tissue types has clear implications not just in neuro-oncology surgery, but for oncology surgery across all specialties.

#### 3.2.5. Patient perspective

Where we have demonstrated the potential merits of our system to clinicians, a key component of the IDEAL 0 checklist is the patient perspective, addressing the question as to whether the technology will be acceptable to patients. This is crucial to establish at an early stage in the development of any new surgical technology, because if it is deemed unacceptable to patients then it will never be translated into practice outside of the research environment.

**Figure 8:**
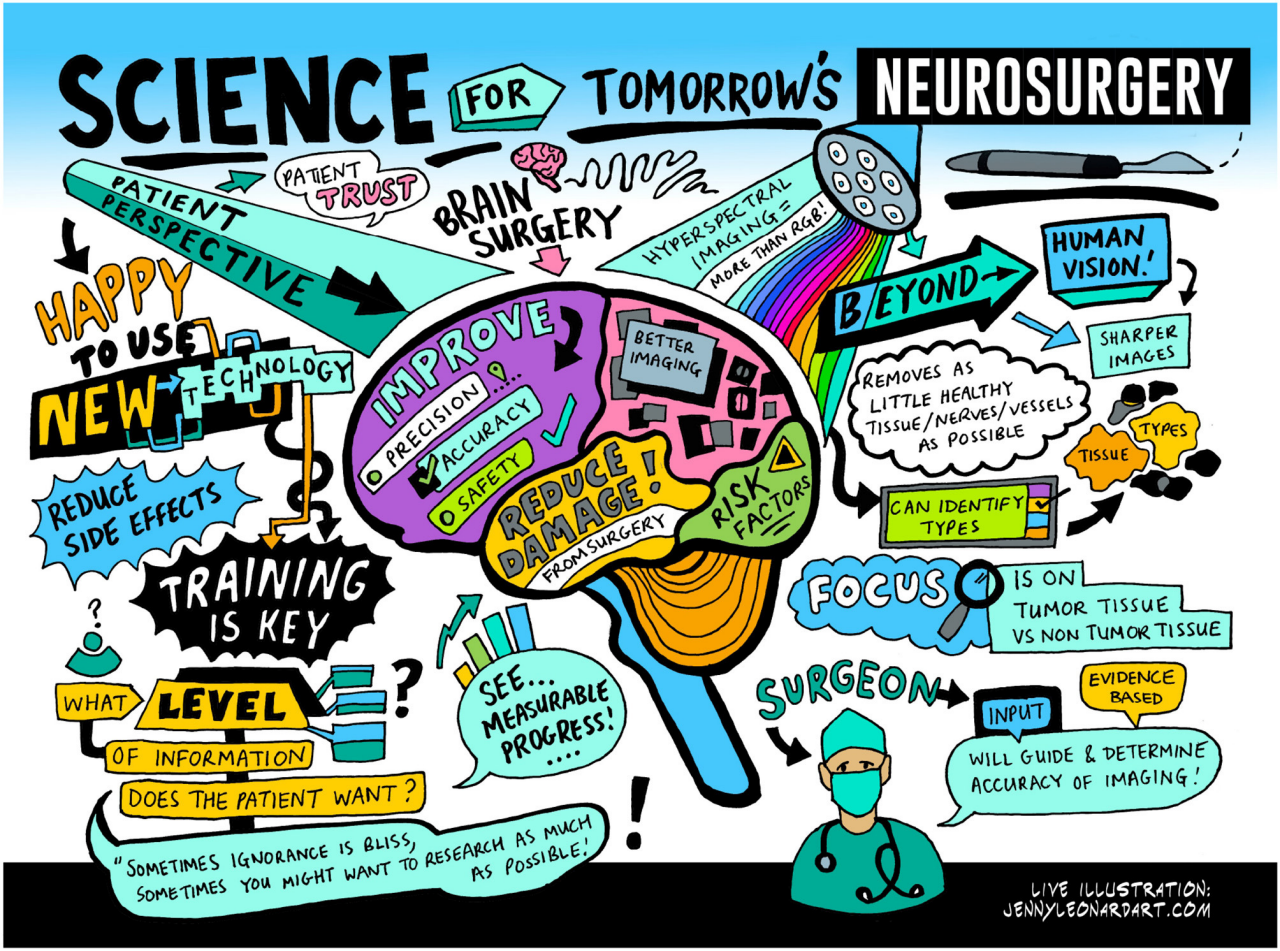
Artwork created from “Science for Tomorrow's Neurosurgery” PPI group meeting providing a visual representation of the proposed technology, its impact and patient/public responses to the technology and its use. Illustration by Jenny Leonard, www.jennyleonardart.com.

To aid the development of this system, a patient and public involvement (PPI) group was formed to explore the use of HSI in neurosurgery. The group was chaired by a patient representative was supported by various patient charities (Headway, The Butterfly AVM charity, The Brain Tumor Charity and The Brain Charity) and comprised 6–10 neurosurgery patients from the United Kingdom. The PPI group provided regular input into the acceptability of the system from a brain tumor and neurovascular patient's perspective and met twice a year via an online platform. Patients provided universally positive feedback on the system, with group participants recognizing that it was “minimally-invasive,” and that they were “happy for surgeons to use new technology,” provided the surgeon was “trained” and had “oversight over any final decisions influenced by the technology.” Each session was attended by a live artist, who captured the key themes of the sessions in an accessible, visual format referencing some of these quotes as shown in Figure 8.

## 4. Clinical “first in human” study (IDEAL 1)

Section 3 demonstrated that our system met the careful safety and feasibility considerations required of a pre-clinical assessment of a new surgical technology. It was thus deemed appropriate to move on to a single case, “first in human” study, following IDEAL 1 guidelines.

### 4.1. Study design

#### 4.1.1. Patient selection

As we were aiming for a proof of concept study and a first in human clinical use of Lightfield HSI, we opted for a single patient study. We decided that a patient with a tumor type that can be readily differentiated by eye would be appropriate as if the system was unsuccessful here, it would not be suitable to take forward to IDEAL 2 (Development and Exploration) studies. The patient needed to be over 18 and able to consent for themselves as well as not having has previous brain surgery, which may add a confounding factor at this stage. Therefore, a patient with a posterior fossa meningioma was selected for this study.

#### 4.1.2. Consent and site

The patient was formally consented for the study (REC reference 22/LO/0046, ClinicalTrials.gov ID NCT05294185) and informed that the use of our Lightfield HSI system would not incur any more than 15 min additional time to their neurosurgical procedure. The study was undertaken at a tertiary neurosurgical referral center in London, UK.

#### 4.1.3. Image acquisition

**Figure 9:**
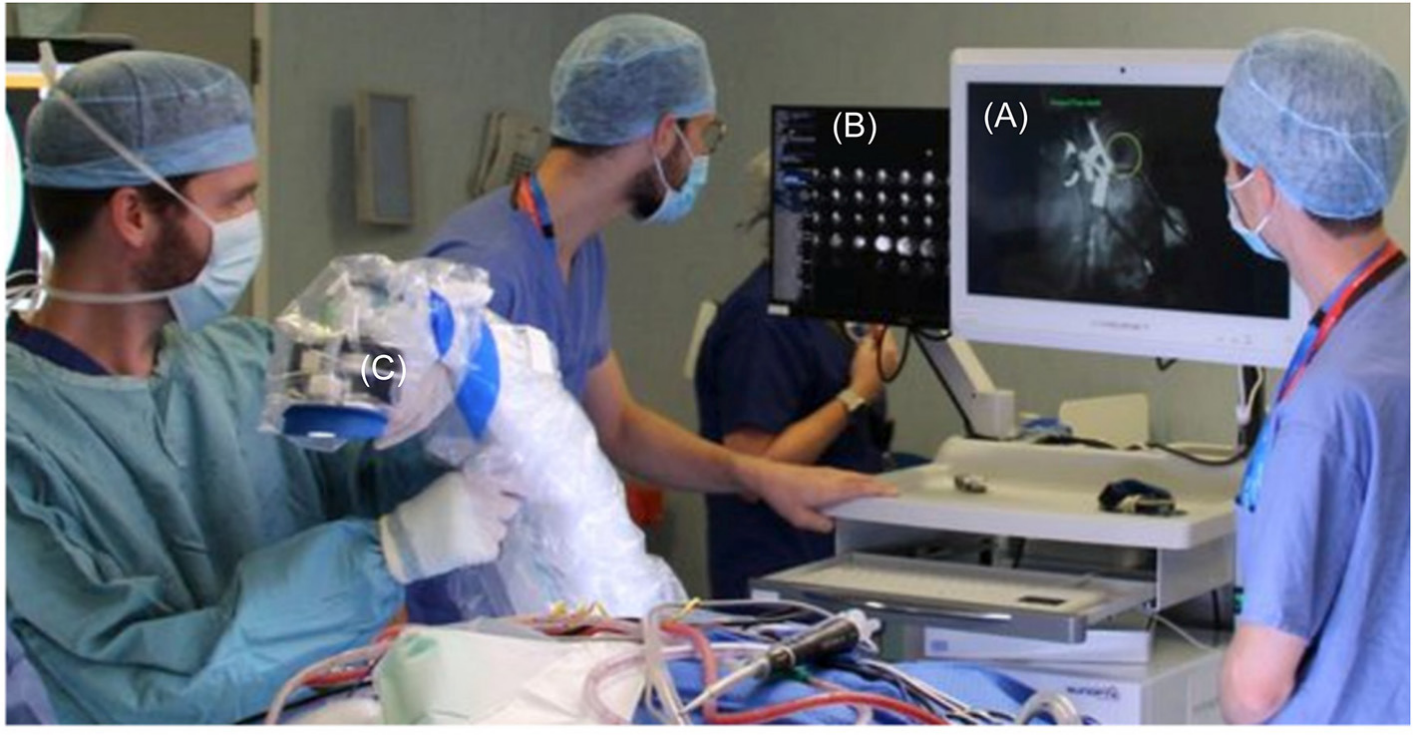
Intra-operative use of lightfield HSI system, draped with Leica 221-88H surgical microscope drape to maintain sterility. **(A)** Low resolution “viewfinder” mode. **(B)** All Lenslet images. **(C)** Draped Lightfield HSI system.

Once the system was draped and ready for use in the neurosurgical operating theater, a white reference was obtained using the Lightfield system in order to facilitate post processing. A clinical member of the research team then liaised with the operating surgeon to identify time points during the operation where it would be appropriate to obtain images. Factors contributing to this were that it was not at a critical stage of the surgery, that imaging conditions were optimal i.e., bleeding controlled and tumor could be visualized and that it was not disruptive to the operating surgeon. During these time points, HSI data was collected using our Lightfield system, as shown in Figure 9, and stored on an encrypted hard drive. White references were taken prior to any imaging in order to account for the lighting conditions and working distance at the time of HSI data acquisition.

#### 4.1.4. Data analysis and interpretation

**Figure 10:**
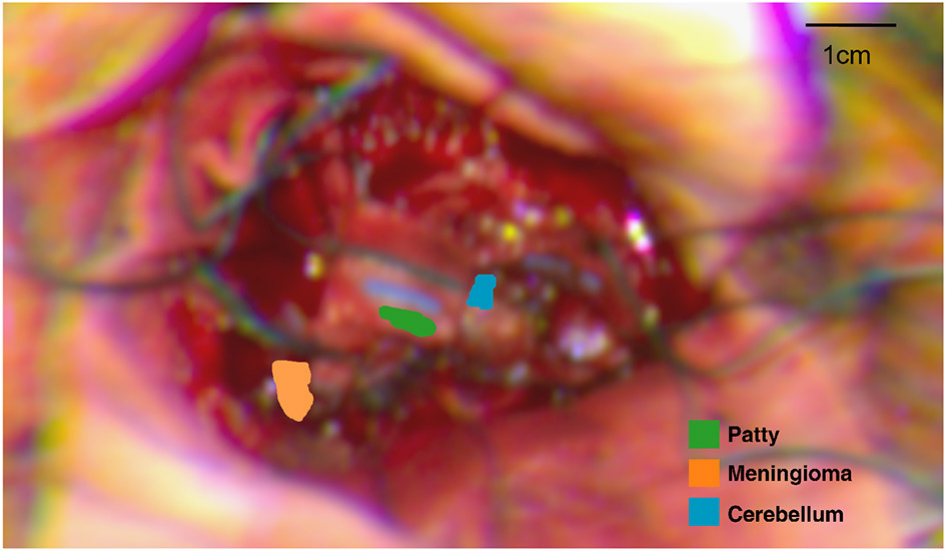
Annotated regions of interest (RoIs) on sRGB image reconstructed from HSI data. Computed distance to target: 27 cm.

**Figure 11:**
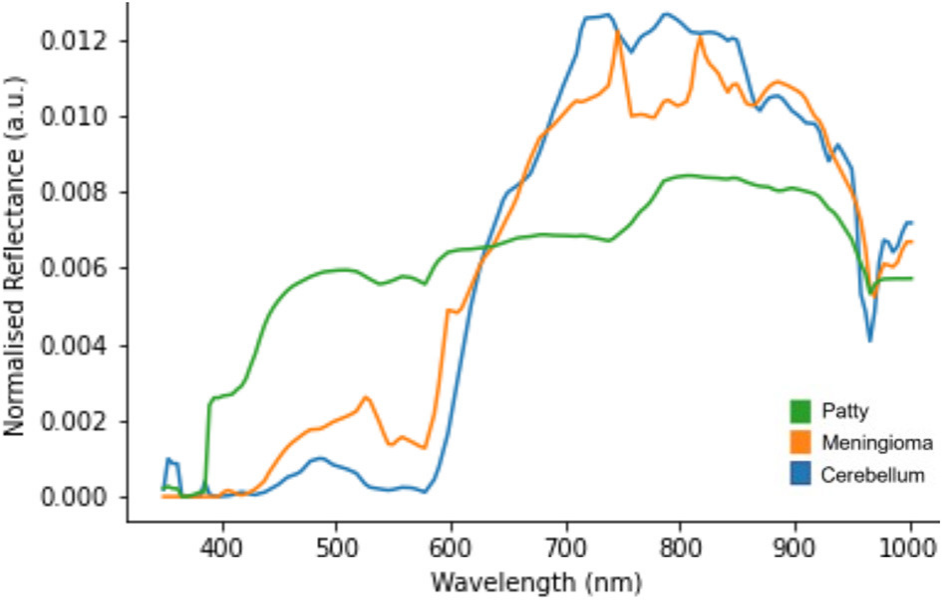
L1 normalized spectral curves of annotated structures as shown in Figure 10.

The raw HSI data was converted into sRGB images using the steps outlined in the pre-clinical section of this work. Specific frames were selected by a clinical member of the research team for image quality and identifiable structure. Each identifiable structure/tissue was then annotated at a pixel level using the ImFusion Labels software as seen in Figure 10. These annotations were used to generate an image mask, where numbers correlating to the annotated structures were assigned to each annotated pixel. These masks were then used to inform accurate pixel selection from the hypercube so that spectral information could then be extracted, and the mean spectrum for each structure was calculated and plotted as seen in Figure 11. A high spatial resolution image captured using the operating microscope at the same time point was used in order to guide annotation.

Figure 11 suggests there is evidence of spectral differences between tissue types (particularly between the 400–600 nm wavelengths), although there is an apparent artifact (most noticeable between 700–900 nm wavelengths) where spectral peaks are not consistent with those typically seen in biological structures. The patty has a markedly significant spectra, as would be expected, although the spectra is not linear (white) likely due to the presence of blood staining.

### 4.2. Qualitative assessment of use

Translation of our system into the neurosurgical workflow is a key outcome of this study and thus a feedback survey for the clinical team to obtain qualitative feedback from the entire operative team was designed. Statements were designed based upon key elements related to the workflow and responses given as a rating of either “strongly disagree,” “disagree,” “neutral,” “agree,” or “strongly agree.” The statements were as follows:

- The system integrated well into the neurosurgical workflow.
- Data capture was straightforward.
- The system was easy to calibrate.
- No disruption to the standard procedure for this operation.

**Figure 12:**
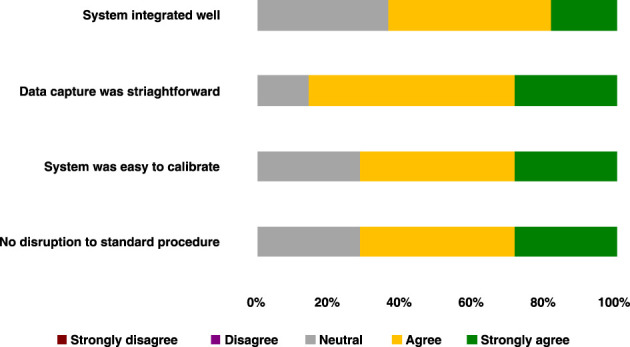
Clinical team survey responses following first-in-human use of our intra-operative lightfield HSI system.

These surveys were completed anonymously shortly after the case by each member of the clinical team in order to reduce bias and ensure recollection of the events of the case remained fresh. No research team members were included in the survey, again to reduce positive bias. 100% of the theater team responded to the survey and this can be seen below, with a total of seven respondents (surgeons = 2, operating department practitioner = 1, anesthetists = 2, theater nurses = 2). Survey results can be seen in Figure 12.

## 5. Toward IDEAL 2a—Development

In this work, we have demonstrated the potential role for Lightfield HSI within neuro-oncological surgery. The limitations seen with the Ultris ×50 system described above indicate that the present system would require improvement in order to become fully integrated into the neurosurgical workflow. We identified a potential solution to some of these issues through mounting and integrating the lightfield HSI system into the neurosurgical operating microscope. This ensured: (1) A mostly static image acquisition environment (surgical action and brain pulsations excluded), meaning that intra-operative image acquisition can be performed based on corresponding microscope images as opposed to the single band gray-scale images used with the Ultris ×50 system; (2) Optimal illumination and focus by utilizing the optics of the operating microscope to ensure the highest quality image acquisition is achieved. Metadata taken from the microscope at the time of imaging can be used to obtain *post-hoc* white and dark references to ensure that quantitative data is obtained.

Whilst integrating a snapshot HSI system into the operating microscope has been demonstrated to be feasible in the literature (Pichette et al., 2016), the size, structure and absence of relay optics with the Ultris ×50 precludes mounting to the neurosurgical microscope. However, very recently, more compact lightfield HSI sensors capable of taking advantage of the advanced relay optics within the Kinevo 900 operating microscope, such as the Cubert SR5, have made this possible, whilst still maintaining a high spectral resolution (51 bands).

**Figure 13:**
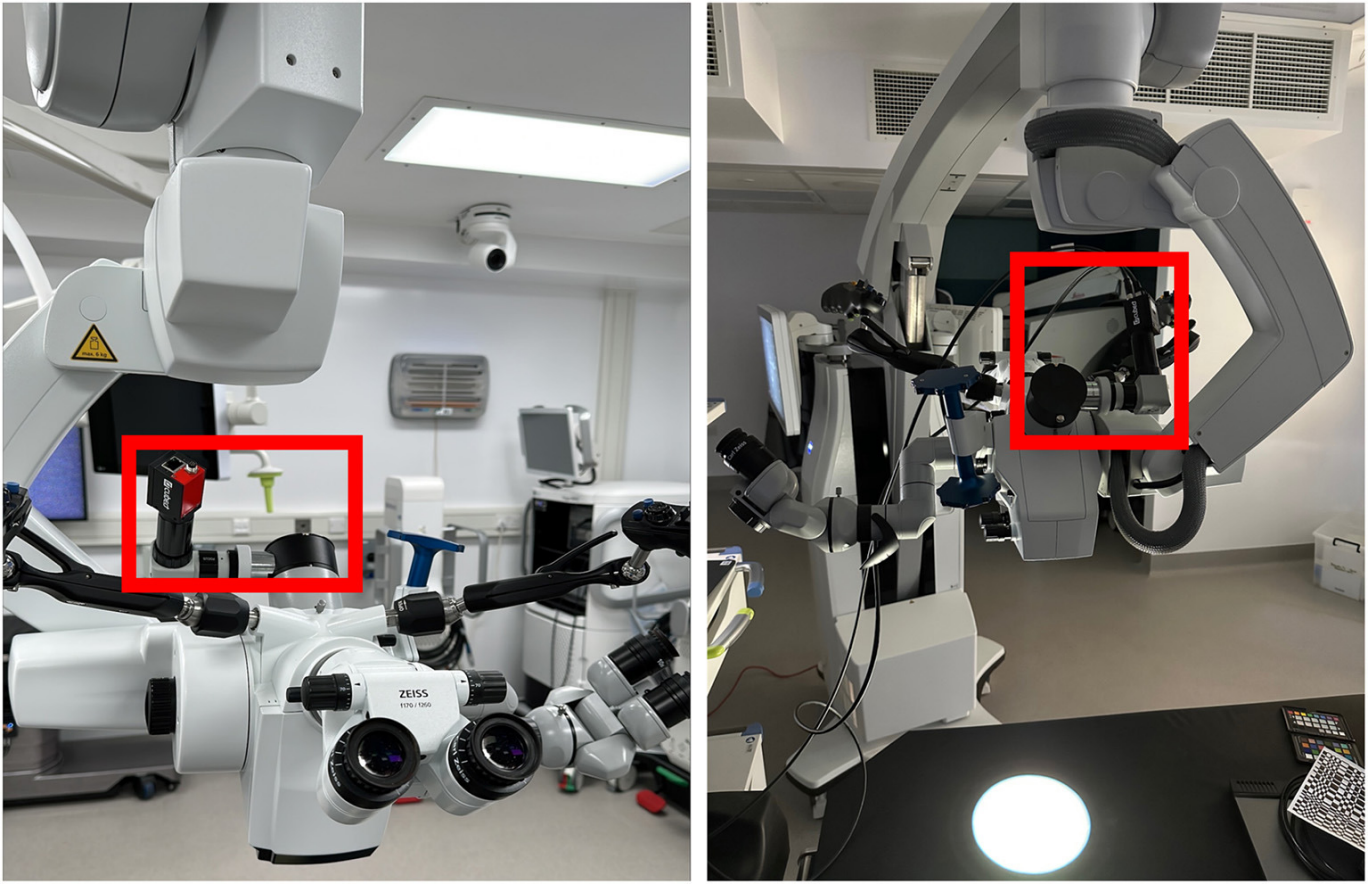
Cubert SR5 Lightfield HSI sensor integrated into the Zeiss Kinevo 900 via means of one-way “beam splitter” (i.e., mirror), camera adapter and Cubert SR5 Lightfield HSI camera.

We have achieved a first integration of the Cubert SR5 by incorporating a commercially available optical mirror in order to direct light from the surgical assistant's port on a Zeiss Kinevo 900 surgical microscope to a threaded camera adapter in order to facilitate mounting of the Cubert SR5 lightfield HSI camera as seen in Figure 13.

**Figure 14:**
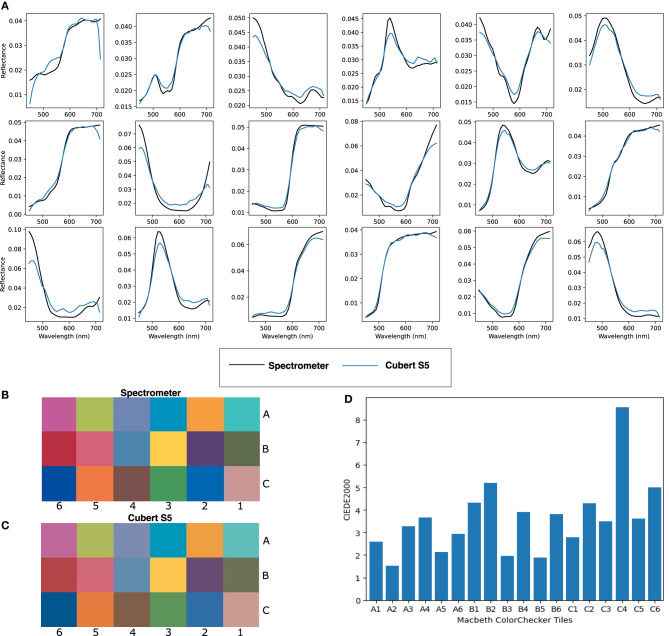
**(A)** Normalized spectra for each corresponding Macbeth ColorChecker tile. **(B)** Ground truth sRGB reconstruction of Macbeth ColorChecker tiles using spectrometer measurements. **(C)** sRGB reconstruction of Macbeth ColorChecker tiles using Cubert SR5 Lightfield HSI integrated with the Kinevo 900 neurosurgical operating microscope. **(D)** CIEDE 2000 errors per tile between spectrometer and Cubert SR5.

Initial color checker data has been acquired in the mock operating room setup and once again demonstrates strong correlation with the known spectra of each tile as seen in Figure 14. It can however be noted that there is a loss of spectral information above 720 nm.

We will continue to focus on the integration of this new set up into the neurosurgical workflow, whilst simultaneously optimizing spatial resolution both computationally and by making use of the inbuilt optical capabilities of the Kinevo 900.

## 6. Discussion

We have demonstrated for the first time that lightfield hyperspectral imaging can be used intra-operatively in order to obtain wide-field, label-free, real-time spectral data from different structures during a neuro-oncology surgical procedure. Our handheld system, allowed this to be done in an ergonomic fashion whilst ensuring sterility was maintained and with a minimal surgical footprint.

The artifact noted between 700–900 nm could be explained by the presence of either specular reflections (where pixels may be disproportionately saturated by a single wavelength band), chromatic artifacts or indeed instability of the Asahi light source in the NIR-IR wavelengths. Artifacts caused by specular reflections may be reduced by automatically removing pixels where a maximum intensity has been reached. However, such thresholding would not counteract chromatic aberrations, which are color artifacts caused by the failure of the system to focus all colors to the same point (Marimont and Wandell, 1994; Leiwe et al., 2021). In our system, the most likely source of chromatic aberation lies in the suboptimal parallax compensation currently used to reconstruct hypercubes. In order to correct for this, the system would require improved algorithms able to handle non-planar objects in order to achieve sharp edge discrimination between structures.

Similarly, when RGB color reconstructions were created from the spectra generated, meningioma and patty appeared intuitively correct. However, cerebellum was far more “red” than would be expected. This is almost certainly due to the presence of blood in the region of annotation, which will skew the average spectra more toward the “red” wavelengths. HSI has a low depth of tissue penetration, meaning the surgical field needs to be kept clear of blood and cerebrospinal fluid, which can interfere with the reflectance measurements. Although important to be aware of, ensuring this would not significantly alter the neurosurgical workflow as this is a necessary step in standard microsurgery to ensure optimal visualization of the surgical field.

It is also noticeable that the RGB image appears more “blurred” than those shown in the pre-clinical testing. This may be contributed to, at least in part, by small amounts of motion blur that come with the use of a handheld system, as well as normal brain pulsations. When considering image clarity, this is best determined by the ability to define edges (van Zwanenberg et al., 2018), a quality that is lacking in the Ultris ×50 sRGB reconstructions, making subsequent annotation of structures difficult.

This lack of clarity may also be secondary to insufficient parallax corrections for this imaging technique. As has been discussed previously, the lightfield HSI is able to obtain a complete hypercube in a single snapshot by utilizing an array of lenslets that all “view” the same object from a slightly different perspective. Therefore, in order to create a single image with good spatial resolution—accurate correction of the parallax effect from multiple different viewpoints is essential.

As well as these above limitations, an appropriate ground truth on a pixel-by-pixel level is challenging to acquire. Addressing this important challenge would be a key goal for any follow-up studies aiming to demonstrate efficacy of the system. This is however beyond the scope of IDEAL 0/1 studies. Nonetheless, the close correlation with spectra obtained from standardized colored reflective tiles with known spectra demonstrates the technical effectiveness of our solution and thus provides an excellent starting point to evaluate its clinical impact.

## 7. Conclusions

This study demonstrated the feasibility and safety of integrating new lightfield hyperspectral imaging technology into the neuro-oncology workflow and obtaining spectra from defined tissue structures. This was achieved with areas for future development clearly identified. This work demonstrates an exciting step forward in the journey to achieving real time, wide field, label free intra-operative tissue differentiation for neruo-oncological surgeries.

## Data availability statement

The original contributions presented in the study are included in the article/supplementary material, further inquiries can be directed to the corresponding authors.

## Ethics statement

The studies involving humans were approved by the London Westminster Research Ethics Committee, REC reference 22/LO/0046. The studies were conducted in accordance with the local legislation and institutional requirements. The participants provided their written informed consent to participate in this study. Ethical approval was not required for the study involving animals in accordance with the local legislation and institutional requirements because all tissues were procured from a butcher which is therefore beyond the scope of the Animals (Scientific Procedures) Act 1986. Written informed consent was obtained from the individual(s) for the publication of any potentially identifiable images or data included in this article.

## Author contributions

OM: prepared the manuscript and was involved in clinical integration of the technology. PN: lead on the software development. MJ: lead on the optical engineering development of the technology. CH: developed software to extract spectra from the hypercube. JQ: assisted in optical engineering development of the technology. MEl: assisted in manuscript preparation and lead the “on the day” use of the technology. AB: assisted in manuscript preparation and data analysis. TT: lead on technical safety. JJ: reviewed the manuscript and contributing to final draft. SP: lead on data recording on the day of surgery. MB: senior optical engineer. KA: clinical lead for the surgical case. SO: reviewed the manuscript, head of school developing technology. MEb: lead from industry perspective in technology development. TV: technical lead for study. JS: clinical lead for study. All authors reviewed and edited the final manuscript.

## Appendix

**Table A1:** Classification of device types following the IDEAL-D framework for device innovation.

**Device classification tiers**			**Examples**	**Regulatory class (EU)**
**Tier 1**	**Tier 2**	**Tier 3**		
**Non-invasive**	**Non-surgical**	Non-invasive devices	*Incision drapes; wound dressings*	*Class I*
			*Tubing used with infusion pump; fridges for storing blood or tissue*	*Class IIa*
			*Dialysis systems; ventilators*	*Class IIb*
			*Organox metra perfusion circuit*	*Class III*
	**Surgical**	Active therapeutic devices intended to administer or exchange energy	*Lithrotripsy devices; surgical ultrasound devices*	*Class IIa or IIb*
**Invasive devices**	**Non-surgical**	Invasive with respect to body orifices	*Indwelling urinary catheters; tracheal tubes*	*Class IIa*
	**Surgical**	Surgical instruments	*Suture needles; staplers*	*Class IIa*
			*Cardio-vascular catheters; external ventricular drains*	*Class III*
		Absorbable surgical implants	*Absorbable sutures*	*Class IIb*
		Non-absorbable surgical implants	*Peripheral vascular grafts and stents*	*Class IIb*
			*Breast implants; total hip replacements*	*Class III*
